# Spirit of the British and American Periodicals, &c.

**Published:** 1842-07-01

**Authors:** 


### Spirit of the British and American Periodicals.

---

### Extinction of Human Races.

Dr. Prichard read a paper on this subject in the Zoological Section of the British Association, which has been printed in a separate form. After alluding to some ancient depopulation, Dr. Prichard turns to what is going on under our own eyes. Whatever, he says, were the causes which destroyed the ancient tribes, we know to what agency we are to attribute the similar fate of many nations who have perished since the historic age commenced. How many whole races have become extinct during the few centuries which have elapsed since the modern system of colonisation commenced? The Guanches, the numerous people of the Canary Islands, now exist only in their mummies, and in the traces of those arts by which they sought to procure for the dead an eternal repose, and which have proved the occasion of transporting their mummies into the museums of Europe. It would be endless to recount the names of tribes and whole nations in America who have been extirpated by the Spanish conqueror of that country. The last race that was utterly destroyed were the Charreas, of whom Dr. P. saw three surviving individuals brought to Paris and exhibited as curiosities. They were a most singular race of people, whose moral and physical character is briefly sketched by Don Felix de Azava, but of whom we have no satisfactory account, nor can we now ever hope to obtain it. The whole country now occupied by civilised nations in the New World was three or four centuries ago thickly peopled by native tribes. A similar process of extermination has been pursued for ages in South Africa, formerly the abode of numerous pastoral nations of Hottentots, a peaceable and inoffensive race, who wandered about with numerous flocks in a state of primitive simplicity, and whose descendants are now found in the miserable and destitute bushmen, condemned to feed upon vermin and reptiles, and rendered savage and cruel by the wretchedness which their Christian conquerors have entailed upon them. Wherever Europeans have settled, their arrival has been the harbinger of extermination to the native tribes. Whenever the simple pastoral tribes come into relations with the more civilised agricultural nations, the allotted time of their destruction is at hand; and this seems to have been the case from the time when the first shepherd fell by the hand of the first tiller of the soil.

To remedy this crying injustice the "Aboriginal Society" has been instituted. It numbers amongst its members individuals of great worth, eminent abilities, and no mean station. Its objects are laudable, and its proceedings praiseworthy. An essay on "The Practicability of Civilising Aboriginal Populations," may be said to represent its views.

In this paper the irreclaimability of the Indians is contested upon these grounds:—

First: Other races, amongst which, for centuries, there existed a want of civilisation, very similar to that which has prevailed amongst the North American Indians, have successfully struggled for their own advancement, and, under the development which cultivation has produced, have reached the greatest height to which human talent and industry have yet attained.

Secondly: We need not trust to this analogy, however strong, to sustain our hope that the Indians, if also favoured by circumstances, might similarly rise. We have numerous scattered examples of Indians of various tribes, which prove that the Indian mind and intellect form no exception to the law of progressive improvement which applies to the human mind generally.

Thirdly: If we proceed from individuals to assembled multitudes, we shall find, that though the opportunities have been few, and invariably very imperfect, they have, notwithstanding the disadvantageous circumstances which attended them, been amply sufficient to remove every doubt that the Indian race has all the requisite capabilities for a thoroughly organised system of social order.

Fourthly: The objection, that notwithstanding these encouraging instances, the result of past experience has generally been of a gloomy and disheartening character, may be met by evidence which shows that there have been powerful and adverse causes in operation, quite adequate to account for all the obstructed good and active evil which we have so much reason to lament, and which we are so loudly called upon to retrieve.

Fifthly: If we look to those instances in which the scheme of removal, suggested by the erroneous principle in question, has been adopted, it will be found that they have invariably been marked as total failures; and that, so far from conferring the advantages which they vainly promised, they have proved amongst the most active means for promoting the spoliation and ruin of the people.

Sixthly: It may be shown that, if the malign causes which have attached so foul a blot on the character of Europeans and their descendants in their treatment of the aborigines of America were suspended, and an opposite feeling substituted in their room, measures, neither expensive in themselves nor doubtful in their results, might easily be adopted, which would confer lasting honour on the authorities by whom they may be introduced, and great and reciprocal blessings both on the red and the white population.

We need not go into the reasoning which, up to a certain point, seems to us conclusive. It is, we think, pretty evident, first, that the experiment of civilising the Indians has hitherto miscarried—secondly, that the Indians have not had fair play—and, thirdly, that, unless a great change occurs, their race will surely be exterminated.

The present system appears to be to induce or force the Indian to retire farther and farther into the forest. A community *settles up* to a native tribe. Its possessions are coveted. The tribe, by fair means or foul, is then induced to *remove*, the land of promise and of exile tempting no European but the fur-trader to seek it. Let the pamphlet speak to the results.

"If the Indians are removed from the neighbourhood of civilised men, will they meet with that abundance of game which was known to exist in the American forests before the European axe and the European rifle were heard amongst them? We have the testimony of various travellers to prove that this is not the case. Every credible evidence confirms the fact, that the game, especially of the larger species, has become more scarce and more wild, and consequently less attainable by those means which the Indians, unaided by European arts, can employ.

If the Indians be removed from the vicinity of their white friends, and driven to encroach upon the territories of other Indians, can we be sure that they will find those Indians ready to receive them as friends, and not regard them as pernicious intruders? Our past acquaintance with the Indian race has shown that the different tribes are very ready to engage in war with each other; that trespasses upon their respective hunting grounds have proved powerful causes of animosity. It is also well known that their wars are of a cruel and exterminating character; and it must be obvious, that if one of the contending parties can command the exclusive or much more general possession of European arms, the total defeat of the other must be certain.

If the Indians, whilst living upon their reservations, surrounded by settlers in some degree advanced in civilisation, and whilst more or less assisted by the superintendence, instruction, and counsel of those who sincerely desire their preservation and improvement, have, notwithstanding, felt the baneful influence of those whose interest it is to injure and demoralise them, can we suppose that, when removed from such protection, they will not be followed by those whose interest in injuring will remain the same, and who will be freed from all the restraints which may hitherto have been put upon their base designs? The fact that every part of the wilds of North America, between the most western settlements of Canada and the United States to the shores of the Pacific, are traversed by the traders of England and America, can leave us little room to hope that a spot can be found upon which the red man may be placed in security against the approaches and contaminations of his white enemies."

"A comparison of the descriprions given of the Indians to the west and northwest, in the narratives of the expeditions of Sir John Franklin and Captain Back, will convince us that, in the interval which elapsed between the two expeditions, the condition of the remote Indians has been rapidly declining. Disease, intemperance, famine, and mutual hostilities, have fearfully reduced the numbers of various tribes. It was unquestionably proved, during the last expedition, that by the irregular movements of the game, or from want of the ammunition by which it was to be taken, the Indians, but more especially the aged, the young, the infirm, and the females, are liable to be reduced to such a state of extreme starvation as to be driven to devour the tattered and filthy skins which they wear as an imperfect protection against the severity of a winter in which the thermometer sinks many degrees below zero—whole families are cut off by the combined influence of cold and hunger, and even cannibalism is known to have been occasionally resorted to under the pinchings of famine."

The Association propose:—1st, To give to the Indians within our provinces, in conjunction with some special but temporary protection, the fullest participation in the rights of British North American subjects, and the permanent security of their landed possessions. 2dly, That all the efficient means at present in existence for the advancement of the Indians in religion, school education, and the arts of civilised life, should be encouraged, improved, and extended. 3dly, That efforts should be made to bring forward and encourage successful and meritorious individuals, in the first place, by employing the system of *concours*, or competition, in the schools, and also amongst those who apply themselves to agriculture and the arts; and, secondly, by appointing the individuals so selected as teachers to be distributed amongst their brethren; and in case of the discovery of more than ordinary talents, by giving them the advantages of a higher degree of instruction in the best colonial schools, or even in this country. 4thly, To form, with the assistance of the most worthy and influential chiefs, such a system of police or civil government as may both suit the Indian character, and lead to the Indian settlements becoming integral parts of the province in which they exist, like the Welsh counties in England and the Basque Provinces in France and Spain. 5thly, To extend the advantages of organisation, instruction, and participation in British laws and British protection, to the Indians beyond our frontier. They will thus not only be put in the way of promoting their own security and happiness, but will also become of incalculably greater importance to us as allies, or rather as fellow-subjects, for the maintenance of our frontier, and the extension of our commerce.

We can conceive no *just* objection to these views or propositions. It is no costly experiment either of property or human life that is suggested. It is a fair trial of the capabilities of Indian character under favourable circumstances, and right auspices. And, unquestionably neither right nor favour has been hitherto extended to the aborigines.

But we confess we are not sanguine of the results. The land of the Indian is coveted by the white man, and the constant stream of emigration from Europe threatens to flow over every tract of the New world, not utterly and hopelessly desolate. What chance the Indian, tutored or untutored, has of avoiding being whelmed beneath it, we will not venture to determine, though we fear it is but light indeed.

---

### PHYSIC IN CHINA AND DIET IN INDIA.

The Lancet* contains an interesting notice of our friend Mr. Martin's Report on the State of the Natives in Calcutta, and a Paper by Mr. Downing on the Theory and Practice of Medicine in China. We shall merely make a *mem* or two from each.

*State of the Faculty in China.*—The Chinese medical men are by no means a privileged class; no examination or diploma is required; any one who has read a few medical books may practise, and the government takes no notice of him, unless he kills people in a manner different from that which the law directs. Doctors, therefore, abound in all the principal cities and towns, and every petty village has its practitioners. The fees are extremely small, except in certain instances where persons have obtained reputation by repeated success. The quacks and mountebanks are in as great abundance as in the most civilised nation of Europe. No encouragement is given to the study of medicine by the government: no schools of public instruction are in existence. In the capital alone a medical board is established, for the express purpose of watching over the health of the members of the royal family. Dispensaries are, however, to be found in the principal cities, where the poor receive gratuitous medical aid from doctors in the pay of the state. In many inveterate diseases the physicians refuse to wait upon the patients, because the disorder is declared, by the rules of practice, incurable. Whenever a sick person cannot eat rice, the physician gives up his case as hopeless; and the luckless practitioner comforts himself with the native adage, "that there is medicine for sickness, but none for fate."

So the medical profession is not more protected in China than in this country. The same political economy would seem to reign in both. A free trade in physic is the guiding principle of the Legislators in either.

*The Ecole Physiologique in China.*—"The human body," so teaches the immortal work, *Ching che chum Ching*, "is composed of water, fire, wood, metal, and earth; the five elements which constitute the substance of everything. As long as the equilibrium between them is maintained, people enjoy health; as soon as one becomes predominant, sickness ensues. All diseases arise from disturbing the natural relation of these component parts, and the art of healing consists in restoring their natural relation. A physician ought, therefore, first to ascertain which of the elements has gained the ascendancy over the others, and, after mature deliberation, he should endeavour to counteract its baneful effects. Inflammatory diseases arise from the prevalence of fire. Distorted eyes and mouth arise from the prevalence of wood over the metals, which contracts the muscles. Under such circumstances earth discharges its nature, its power relaxes in the interstices, the eyes become hollow, and the muscles are contorted, as may be abundantly proved from the classics."

*Chinese acumen in feeling the Pulse.*—A great many doctors amongst us can do a great deal by feeling the pulse. They can dispense with the use of their ears, and laugh at the stethoscope, yet we doubt if many of them are equal, after all, to the Chinese. The physicians of the Celestial Empire say they can distinguish by the touch no less than twenty-four different kinds of pulse; such as

---

* March 5, 1842.

the hard, soft, wiry, intermittent; and assert that each part of the body has a pulse peculiar to itself alone, and in relation to some distant organ of the body. Thus there are three pulses in the arm, called the inch, the bar, and the cubit; and the pulse at the wrist of the left hand is in accordance with that of the heart, while the pulse of the liver is to be found higher up in the same extremity; and the pulses of the stomach and of the lungs are to be found in the same positions in the right arm. By merely feeling the pulse in different parts of the body they can ascertain the seat of a disease, with the symptoms and mode of cure. They moreover, pretend that they can discover in this way, whether or not women will be blessed with children, whether the offspring will be of the male or female sex, and other equally obscure, but interesting, matters of inquiry.

*Chinese Pathology.*—The great work Ching che chum Ching lets us into this secret. Diseases are simply yet satisfactorily classified thus:—

1. Violent and mortal fits, divided according to the causes which have induced them; as wind, cold, heat, moisture, vapour, nutriment, &c.; also suicide and accidents.
2. Indispositions occasioned by heat, moisture, dryness, eating and drinking, fatigue, &c.
3. Fevers and agues; hot, moist, dry, malignant, cold.
4. Defects in respiration, suffocation, short breath, dropsy, cough.
5. Vomiting of phlegm, pus, green and sour water.
6. Hæmorrhages; bleeding at the nose, tongue, teeth, and ears; vomiting and coughing of blood.
7. Pains in the heart, head, face, stomach, &c.
8. Paralytic complaints, gout, acute rheumatism.
9. Rheumatic complaints of seven different kinds.
10. Mental disorders, insanity, madness, immoderate laughing, fits of fear, rage, trembling, &c.
11. Sundry diseases, including immoderate perspiration, sleeplessness, somnolency, lassitude, yawning, &c.
12. Diseases of the viscera, diarrhœa, dysentery, &c.
13. Ophthalmic complaints.
14. Pains in the ear, nose, tooth, mouth, jaw-bones; and also cutaneous diseases, and those of the hair.

Turn we from the Celestial Empire to *Hindostan.*

We often hear of the advantages of low diet, from those who would persuade or force their neighbours to resort to it. Poor-law commissioners, boards of guardians, &c. think Hindostan an earthly Paradise. *There* the poor enjoy the beau ideal of a pauper's diet, as will at once be seen.

A man, his wife, and two children, eat two maunds of rice, which cost a rupee (2s.) in the month; if very poor, they only get with it herbs gathered in the fields, and green pepper pods; salt and a little oil are procured by those in somewhat better circumstances; the middling classes eat split peas, greens, fish, &c. The ashes of various plants are often substituted for salt by the poorest of the people. What is the effect of this miserable dietary, to which some speculators wish to reduce the hard-working people of England? "Whoever has travelled much with the natives," says Dr. Hamilton, "and been witness to the *weakness of their constitution* in resisting the changes of air or water, will agree with me in saying, that those who enjoy a diet which includes animal food and strong liquors in moderate quantities, are best able to resist the influence of unhealthy climates, and the sudden change of air." "It has always appeared (to Mr. Martin) a *great mistake* to view the diet of the Bengalee as *prescribed by climate:* on the contrary, he believes it to be far below the standard required for support under all the changes of the seasons: in the hot weather and rains, it is not sufficient to supply the great waste; and in the cold weather, *its poverty is alike injurious to health.*" The Bengalees are inveterately idle, and will not or cannot work hard; they earn a penny, threehalf-pence, or two-pence a-day. The Mahomedans have a better system of diet; they are, therefore, on the whole, more robust, and more capable of sustaining efforts than the Hindoos. From a calculation of Dr. Duncan Stewart, it would appear that for six years the average annual mortality of the Hindoo population in Calcutta was *one* in 17⅓; while in the better fed and clothed Mahomedan population, only one in 38½ died! What have the vegetable and starvation doctors to oppose to that "remarkable fact?"

---

### Contraction of the Flexor Muscles of the Thumb, or "Scrivener's Spasm" cured by Division.*

The chief characteristic of this affection is an absolute incapability of using the pen, for writing, although the strength and motions of the hand remain unimpaired for all other purposes. Some authors regard it as a species of spasm; others, as depending on paralysis; it is generally permanent, but occasionally appears at intervals, and then is commonly brought on by long-continued use of the pen. This affection, though apparently slight, is most obstinate, and resists every method of treatment that has hitherto been employed against it.

M. Stromeyer, in one case, ineffectually divided the short muscles of the thumb. The patient refused any farther operation.

The subject of the next case had suffered under the disease for fifteen years, when first seen by M. Stromeyer. The rigidity of the muscles of the ball of the thumb was not, however, present, but the last phalanx of the thumb became suddenly flexed, whenever the patient attempted to write or play on the piano.

The long flexor was not permanently contracted, nor did it impede any other motions of the hand. From the deep situation of the muscle it was not easy to divide it separately. M. Stromeyer bent the first phalanx strongly to a right angle, and at the same time turned the thumb as much out as possible; he then passed a very small, curved tenotome underneath the tendon, and divided it. The sensibility of the thumb was very considerably diminished after the operation, but was restored, on the dorsal aspect the next day, and on the palmar aspect within a fortnight. The natural power of moving the thumb, also, returned at the latter period, and the patient was able to write or play on the piano without the slightest return of the spasm.

---

### Iodine Injections for Hydrocele.†

It appears that Mr. Walne has been trying this method, proposed originally by our Indian brethren. He observes:—Those parts of the instruments used which are metallic require to be guarded against the action of the iodine, by being carefully oiled beforehand, and freed from what may remain upon them, by being dipped into a solution of potassa immediately after being used. If some care of this kind be not bestowed they will quickly be corroded and injured, the affinity of iodine for metallic substances being very strong, and its effect destructive.

---

* Provincial Medical and Surgical Journal. Feb. 5, 1842.

† Medical Gazette. March 11, 1842.

But a small quantity of fluid is required, and the apparatus may be advantageously much reduced in size. As to the trocar, I have, with great comfort to myself and my patients, for many years used one of not more than a third of the size employed by some surgeons. From 6 to 12 drachms of the following injection, the ingredients being mixed just before the operation is performed, is a sufficient quantity, and it is not material, I think, whether warm or cold water be used, though I commonly have it warm.

℞ Tr. Iodin. ʒj. ad ʒij.; Aquæ tepid. ʒx. M.

When introduced, this should be moved about in the tunica vaginalis by gentle handling, and after from three to five minutes may be allowed to escape. It produces some uneasiness at the time, but not enough to prevent a patient walking, perhaps a mile or more, immediately after the operation, with the part suspended.

---

### Formula for the Infusion of Chirayta.

Mr. Houlton has long been in the habit of preparing this. He gives this formula.

Infusum Chiraytæ. ℞ Herbæ Chiraytæ, ʒss.; Aquæ ferventis ℥xvj. Macera per horas duas et cola.

This I find sufficiently strong, and it is a very valuable simple bitter: I prefer it to any we have in our Pharmacopœia. Its efficacy in a case of chronic bronchitis in an aged person whom I attended a few months ago was very decided: he rapidly recovered under its use, though this was a very severe case: the expectoration was very great, and the powers of the system very low.

There is one circumstance worth notice, which is, that this infusion will keep for a considerable time without undergoing any sensible change. It has kept good for six months during the summer months in a green-stoppered bottle.

Mr. Houlton gives a formula for a Tincture of Chirayta long followed by Reece and Co. of Piccadilly.

Tinctura Chiraytæ. ℞ Herbæ Chiraytæ, ℥ij.; Sassafras Concisæ, ʒiij.; Pterocarpi Concisi, ʒij.; Spiritûs Tenuioris, f℥xxiv. Macera per dies quatuordecim et cola.

---

### Pharmacy in Germany.

A good sketch of its present state, or, rather of the present state of its professors, is given in the last Number of the Pharmaceutical Journal.

The Apothecaries or Pharmaceutical Chemists in Germany are officers of the government; and the laws which regulate their education and license to practise Pharmacy, are such as cannot fail to secure to the public the advantage of first-rate talent, and to those who have acquired this qualification, a just and adequate reward.

Those who are designed for the profession are obliged, before they become Apprentices, to undergo an examination before a commission of Pharmacy, in French, Latin, Greek, elementary Mathematics, and other branches of general knowledge. The usual term of apprenticeship is three or four years; during which period every opportunity is afforded for studying the processes in the laboratory, and the science of Chemistry, as well as the usual routine of manipulation in the shop.

At the close of his apprenticeship the student commences his labours in the university, which generally occupy about two or three years. The subjects which chiefly engage his attention are, Physics, Mathematics, Botany, Zoology, Mineralogy, Ghemistry, and Pharmacology. In some cases, Anatomy and Pysiology are included; and a laboratory is attached to the university where the pupil may attend for a year or more if he feel disposed. But there is no definite curriculum prescribed by law. The student is at liberty to use his own discretion as to the manner in which he obtains his knowledge; but the rigour of the examination is a sufficient security against idleness or inattention. The subjects on which proficiency is required being stated, there is no excuse for neglecting any thing which is important.

The colleges in Germany are celebrated for the facilities which they afford for the acquisition of knowledge; and being under the immediate superintendence of government, and possessing ample revenues, the advantages of a complete education may be obtained at a very moderate expense.

A majority of the leading Apothecaries take the degree of Doctor in Philosophy; for which purpose they attend the lectures of the Professors of the Philosophical Faculty.

This distinction, however, is not necessary; and the examination for licence to practise, is confined to those physical and natural sciences which have been above mentioned.

A candidate who is proved to be an immoral character is not allowed to pass an examination; or, if he has already passed, he is prohibited from practising as an Apothecary.

Having devoted six or seven years to the study of his profession, and having passed the requisite ordeal, the Apothecary in Germany finds himself in a peculiar dilemma. He has proved himself proficient in all the practical details of his business; he has received his licence to practise Pharmacy, and he is possessed of the means of taking or opening a shop.

In England this would be an easy matter. He might, in the course of a few days, find himself standing behind his own counter, supplied with every requisite for carrying on a prosperous business, except one most important item—namely, customers. Unless he happen to possess the advantage of a large circle of friends and connexions, he might wait many years for a favourable turn in his fortune, and ultimately have cause to regret having embraced a profession already overflowing with numbers, and in which the majority can with difficulty gain even a subsistence..

But in Germany the case is very different. The number of Apothecaries being limited, the only difficulty, after a licence has been obtained, consists in meeting with a shop. This obstacle having been surmounted, anxiety is at an end, as every Apothecary enjoys ample scope for the exercise of his abilities—is protected from ruinous competition, and cannot be displaced, excepting in the event of any misdemeanour on his own part.

The average number of Apothecaries in Germany, is one to a population of 5000. In this calculation, the inhabitants of neighbouring villages are included. As the population of any place increases, and the wants of the public require an additional number of Apothecaries, permission is granted by the government to such candidates as stand first on the list, and this is the only way in which a shop can be obtained, except in the event of the death or removal of an Apothecary already in business.

The heirs of an Apothecary deceased, are at liberty to dispose of the concern as they may think proper, provided the purchaser or successor be qualified by licence to practise Pharmacy.

No competition in prices is allowed in Germany, and a price list is monthly published by authority in order to ensure uniformity. The scale of profits is higher than could be obtained in this country, and any deviation is strictly prohibited, unless a certificate be produced signed by a Physician and a Magistrate, stating that the party is unable to pay the usual price, in which case the Apothecary is allowed to use his own discretion. Town paupers are supplied at a discount of 30 per cent., which is allowed by the town.

The law is equally severe against the adulteration of drugs, or the sale of such as are of inferior quality, and every shop is triennially subjected to a rigid examination by officers appointed for the purpose, comprising a Medical Assessor, a Physician, a Police officer, and an Apothecary from a different town or district. In case of suspicion, a shop may be visited at any time, at the discretion of the above officers. The report of this examination contains minute particulars on every subject connected with the nature and condition of the stock, the number of pupils, the books and apparatus, and any incidental matters which may attract attention. No Apothecary is allowed to keep more Apprentices than Assistants.

The establishment of an Apothecary is required to comprise a shop (called (*officin* or *apotheca*), a laboratory, store-rooms, and cellars, and a separate room for poisons. Each store-room or cellar must be under lock and key, and must be provided with an inventory of its contents.

The stock of herbs must be renewed annually, except foreign plants, which are to be replaced once in two years.

On the first occasion of any culpable neglect or deficiency, the delinquent is severely reprimanded: the second offence is visited with a fine, the amount of which is regulated by the circumstances of the case, and is modified by the degree of importance of the shop: if the misdemeanour be repeated a third time, the shop is confiscated, the licence removed, and the party is prohibited from following the business at any future time. In this case he is allowed to sell the concern, and this is not a difficult matter, as there are always a considerable number of licensed Apothecaries waiting for such a chance.

The price of a shop, including stock and fixtures, varies from £600 to £1400, in the towns of Germany; in large cities, an establishment is not unfrequently worth £7000 or £8000, and in some cases, they have been known to realize as much as £15,000 or 100,000 dollars. The income bears a corresponding proportion, but is in all cases considerable, and fully adequate to the wants and comfort of the Apothecary.

In case of the illness or absence of an Apothecary, he is obliged to have a *provisor*, or confidential superintendent, on whom devolves the responsibility of the business.

An Apothecary who relinquishes his profession for the space of five years, and afterwards wishes to resume it, is required to pass a new examination.

The practice of Medicine being distinct from that of Pharmacy, the Apothecary is not allowed to visit patients; but his duties consist in selling and preparing drugs, and compounding prescriptions.

Prescriptions of a powerful nature or poisonous quality are not allowed to be repeated without a fresh order from the medical attendant, and all external applications must be labelled *external*, and distinguished by a blue label.

No drastics, emetics, diuretics, or emmenagogues can be retailed except by direction of a medical practitioner; and the Apothecary is expected to satisfy himself respecting the legal qualification of the prescriber, and thus becomes responsible for every prescription which is prepared at his shop.*

It is obvious that such a state of things may suit a country like Germany, where Government has a finger in every pie, and almost regulates the hour at which a man may go to the water-closet. But to our Island with its commercial republicanism it is totally inapplicable. Here free trade obtains in quackery at all events, if not in other things, and any man may set up to physic or to poison his neighbours, provided his neighbours of their own choice and inclination determine to be poisoned or physicked by him. Each system has its advantages

---

* Pharmaceutical Journal. March, 1842.

and evils. We doubt whether, on the whole, our's is not the best, at least for our *redundant population.*

---

### BLISTERING PLAISTER.*

According to Dr. Müller, the uncertainty which sometimes attends the effects of blistering plaster, as usually prepared, may be ascribed to the circumstance of the vesicating principle remaining locked up in the tissues of the fly.

In order to obtain a plaster more uniform in its operation, Dr. Müller recommends that the cantharides be left to digest in the plaster, kept fluid at a moderate heat, for five or six hours.

I consider this suggestion of Dr. Müller's a very good one to follow: it nearly corresponds with what M. Guibourt has said on the same subject; but the prolonged digestion of the cantharides ensures the solution of the active principle more effectually than would be the case if they were merely incorporated with the plaster while still hot, according to M. Guibourt's recommendation.—*Journal de Pharmacie.*

---

### FINE WORDS IN MEDICINE.

The Lancet of the 26th of March, 1842, contains the following very sensible letter, which, as it is addressed to ourselves, we insert.

*To the Editor of* THE LANCET.

SIR,—Will you permit me to present to the editors of the "Medico-Chirurgical Review" a few remarks on the style of the medical literature of the present day, with which I think they will agree. We laugh at the Americanisms, as we term them, which we see copied from the Yankee newspapers, but as great faults are daily committed among our own medical authors. Many of the writers in our periodicals, and even in some of our larger works, seem to have their heads so filled with French and German words and phrases, that they have quite forgotten their native English. Some there are, too, who affect the use of strange words derived or adopted from the learned languages, wishing apparently to impress us with the idea that they converse so much with the ancients, that they have forgotten that they live in these degenerate days. Now all this appears to me to be either mere affectation or excessive carelessness. There is nothing almost in all that we medical men have to say, which cannot be expressed in the copious stores of our own language. Let me give you a few examples.

In a late excellent work on the ear, three species of deafness are distinguished, as, kophosis, paracousia, and dysecœa. The first two I comprehended at once, but I had fairly to turn over my Greek Lexicon before I could get to the bottom (or root) of the last.

Some of our Irish brethren are particularly fond of this sort of thing, using outlandish expressions. Thus, one man uses the French word *chronicity* for *duration,* an opposite *sense* for an opposite direction; another speaks of a pulse of a *dicrotous* character; a third talks of *consensual* actions, instead of consentaneous, and of *retro-peritoneal* cellular tissue, when he means the tissue behind the peritoneum.

When one man wishes to express the taking of food, he cannot find a shorter way of doing it than the "ingestion of aliment:" and another, in long-winded

---

* Pharmaceutical Journal. March, 1842.

phrase, tells us that his patient "desires to micturate;" another lengthens the shortening of tendons by calling it *contractation;* and a gentleman of considerable ophthalmic reputation cures near-sighted persons of their defect by means of an instrument with the euphonious and sesquipedalian appellation of the *myopodiorthoticon!* One would almost think that these gentlemen agreed with the diplomatist who gave it as his opinion, that "the use of language was to conceal our thoughts."

Hoping that you, the editors above-named, will find room to copy these few lines, and add some remarks of your own, to give them weight:

I am, Gentlemen, your obedient servant,

A PLAIN MAN.

The Plain Man knows that we have always set our face against the barbarous jargon he complains of. It is high time that a stop were put to these coinings. We got on very pleasantly without them, and understood each other and the subject as well when we spoke English, as now when we are talking in Heaven knows what lingo. Nay, we cannot help thinking that the "plain men" both understood and understand the subject better than many of the wordy writers of this new school, who raise such a dust of verbiage that we cannot see their meaning, if they have any.

The rage for manufacturing terms, and over-laying our own terse and manly language, with the periphrastic inflections of the French and German, came in with the Cyclopædia of Practical Medicine.

If the fashion goes on, we shall damage the character of our writings irretrievably. We have some British Classics amongst us—we shall have no more. The works of Pott or Cullen are the works of accomplished gentlemen, embued with the genius of our tongue, and casting a lustre on our literature. The infusion of Norman and Latin and Greek tempers and mellows, while it does not emasculate, the stock of hardy Saxon, and the delighted reader rises from the simple, yet cultivated page, with his fancy engaged and his taste improved. A work of the modern school is of a different kidney. Its big mouthed phrases, long-legged idioms, and awkward progress, remind one of the graces of a May-day lord, mud on his heels, a cocked-hat on his head, tinsel on his rump, and a prigged *wiper* in his hand.

We would not, however, be mistaken. We are far from thinking our language all-sufficient. It has been built up by bits, and its character is essentially composite. But what we complain of, is the reckless and ridiculous coining of new names to express things perfectly known by the old ones—the tampering with the construction of our tongue—and the ignorance or disregard of its essentially concise and Saxon character. This is a serious evil, and unless we can repel the Barbarians, the genius of our literature will sink under their invasion.

---

### POOR LAW MEDICAL RELIEF.—NEW REGULATIONS OF THE POOR-LAW COMMISSIONERS.

The remonstrances of the profession, the decision of a parliamentary committee, and, probably, a sense of justice, tardy though it be, have at last had their effect on the Poor-law Commissioners. Some of the main grievances are to be redressed. Others still remain in abeyance, but enough has been obtained to warrant us in expecting with confidence the ultimate attainment of every just and reasonable claim.

The result of this war with the Poor-law Commissioners reads a significant but painful lesson to both parties. The Commissioners have learnt the futility of a contest with the rights and feelings of the medical profession, and they must tacitly acknowledge the impolicy of their past course. The profession, on the other hand, have been taught the lesson which authority is constantly enforcing, that what is pertinaciously denied to justice must be conceded to clamour. This is the premium upon agitation which modern legislation so liberally holds out. The injured have but to render those in authority uncomfortable, and demands are then conceded which were previously denounced as monstrous.

The profession too have learnt the utility of a medical press. Their journals have carried this question for them. They have been the means of giving publicity to the resolutions of the bold, of encouraging the timid, and uniting all.

But to the regulations.

1. TENDER.

Art. 1.—It shall not be lawful for the board of guardians of any of the said unions, by advertisement, or other public notice, printed or written, to invite tenders for the supply of medicines, or for the medical attendance on any of the paupers within any such union, unless such advertisement or notice shall specify the district or place for which such supply of medicines and such attendance is required, together with the amount of salary or other remuneration fixed or approved by the poor-law commissioners, as the consideration for such supply of medicines and such attendance, or either of them.

Art. 2.—All salaries or other payments to any medical man, fixed by any of the said boards of guardians, and every contract made by any of the said boards of guardians with any medical man, in pursuance of any advertisement, or other notice, inviting medical men to tender their services at a sum or sums not named in such advertisement, or notice, shall be deemed to be fixed or made in opposition to the rules and regulations of the poor-law commissioners in force in this behalf, and all payments made towards such salary, or in fulfilment of such contract, shall be disallowed in the accounts of the parties authorising or making the same.

Art. 3 specifies the qualifications of medical officers. Henceforward every officer must possess one of the four following qualifications:—

1. A diploma from the Royal College of Surgeons in London, together with a degree in medicine from an university in England, legally authorised to grant such degree, or together with a diploma or licence of the Royal College of Physicians of London.
2. A diploma from the Royal College of Surgeons in London, together with a certificate to practise as an apothecary from the Society of Apothecaries of London.
3. A diploma from the Royal College of Surgeons in London, such person having been in actual practice as an apothecary on the 1st of August, 1815.
4. A warrant or commission as surgeon or assistant-surgeon in her Majesty's navy, or as surgeon or assistant-surgeon or apothecary in her Majesty's army, or as surgeon or assistant-surgeon in the service of the Honourable East India Company, dated previous to the 1st of August, 1826.

Art. 4.—Provided always, that if it shall not be practicable for the board of guardians to procure a person residing within or near the district in which he is to act, and duly qualified in one of the four modes recited in Art. 3, to attend on the poor in such district, or that the only person resident in or near such district, and so qualified, shall have been dismissed from office under the seal of the poor-law commissioners, or shall be judged by the poor-law commissioners to be unfit or incompetent to hold the office of medical officer, then and in such case the board of guardians shall cause a special minute to be made and entered on the usual record of their proceedings, stating the reasons which, in their opinion, make it necessary to employ a person not qualified as required by Art. 3, and shall forthwith transmit a copy of such minute to the poor-law commissioners for their consideration; and the poor-law commissioners may, if they think fit so to do, permit the employment by such board of guardians of any person duly licensed to practise as a medical man, although such person shall not be qualified in one of the four modes required by Art 3.

Art. 5.—Provided also, that it shall be lawful for the board of guardians, with the consent of the poor-law commissioners first had and obtained, to continue in office any medical officer duly licensed to practise as a medical man already employed by any such board of guardians, although such medical officer may not be qualified in one of the four modes required by Art. 3.

So the "Tender System" is given up.

2. AREA OF DISTRICTS.

*Maximum Area and Population of Medical Districts.*

Art. 6.—It shall not be lawful for the board of guardians to assign to any medical officer, to be by them hereafter appointed, a district which shall exceed in extent the area of fifteen thousand statute acres, or which shall contain a population exceeding the number of fifteen thousand persons, according to the then last enumeration of the population published by authority of Parliament.

Art. 7.—Provided always, that where any medical officer may, on the day on which this order shall come in force, hold any district exceeding either in area or population the limits fixed in Art. 6, and such medical officer may have been appointed to such district for any time not exceeding twelve calendar months, he shall continue to hold his office, if not otherwise removed therefrom, up to the expiration of the time for which he was so appointed, but that where any medical officer shall have been appointed to any district exceeding the said limits in area or population for any space of time longer than twelve calendar months from the day in which this order shall come into force, the continuance of such officer in his office shall cease and determine on the 25th day of March, 1843, or whenever the term of such appointment may expire, whichever shall first happen.

Art. 8.—Provided also, that if it shall be impracticable for the board of guardians to divide any union into districts containing respectively an area and population less than is specified in Art. 6, then and in such case the board of guardians shall cause a special minute to be made and entered on the usual record of their proceedings, stating the reasons which in their opinion make it necessary to form a district exceeding the said limits, and shall forthwith transmit a copy of such minute to the poor-law commissioners for their consideration, and if the poor-law commissioners shall signify their approval thereof to such guardians, then and in such case, but not otherwise, such guardians may proceed to appoint a medical officer for the said district.

Art. 9.—Provided also, that the limits of fifteen thousand statute acres prescribed in Art. 6, shall not apply or be in force in respect to any medical district situate wholly or in part within the principality of Wales; but no medical district situate wholly or in part within that principality, shall be assigned to any medical officer residing more than seven miles from any part of any parish included within such district, unless the formation of such district shall have been specially sanctioned by the poor-law commissioners in the same manner as is directed in Art. 8.

It will generally, we conceive, be admitted that the maximum assigned to the districts is too great. Yet there is a great improvement on the old system, where caprice was the only limit.

3. REMUNERATION OF MEDICAL OFFICERS.

Art. 10. lays down a rate of remuneration for extra services. No salary of any district medical officer, or contract made by any board of guardians with a district medical officer, shall include the remuneration for the operations and services of the following classes performed by such medical officer in that capacity for any out-door pauper, but such operations and services shall be paid for by the board of guardians, according to the rules specified in this article.

| | | | | |
|---|---|---|---|---|
| 1. Amputations of leg, arm, foot, or hand .. .. .. .. .. | 5 | 0 | 0 |
| 2. The operation for strangulated hernia .. .. .. .. .. | | | |
| 3. The operation of trephining for fractured skull .. .. .. | | | |
| 4. Treatment of compound fractures of the thigh .. .. .. | | | |
| 5. Treatment of compound fractures or compound dislocations of the leg .. .. .. .. .. .. .. .. .. .. | | | |
| 6. Treatment of simple fractures or simple dislocations of the thigh or leg .. .. .. .. .. .. .. .. .. .. | 3 | 0 | 0 |
| 7. Treatment of dislocations or fractures of the arm .. .. .. | 1 | 0 | 0 |

The above rates to include the payment for the supply of all kinds of apparatus and splints.

Provided that in every such case the patient survives the operation not less than thirty-six hours, and that he has required and has received several attendances after the operation by the medical officer, who has performed the same.

Provided also, that except in cases of sudden accident immediately threatening life, no medical officer shall be entitled to receive such remuneration for any amputation, or for the operation of trephining, unless he shall, before performing such amputation or operation, have obtained at his own cost the advice of some member of the Royal College of Surgeons of London, or some fellow or licentiate of the Royal College of Physicians of London, and shall produce to the board of guardians a certificate from such member of the Royal College of Surgeons, or such fellow or licentiate, stating that in his opinion it was right and proper that such amputation or operation should be then performed.

Art. 11.—All trusses furnished by a medical officer in consequence of any contract with or direction of a board of guardians, shall be charged by such medical officer at the cost price, including carriage, and be paid for accordingly by such board of guardians.

Art. 12.—The delivery of any woman in childbirth and the subsequent medical attendance upon her by any medical officer, in that capacity, whether in or out of the workhouse, shall be paid for by the board of guardians in the manner specified in this and the following article—that is to say, in cases in which any such medical officer shall be called on by order of any person legally qualified to make such order, to attend any woman in or immediately after childbirth, or shall be required, under circumstances of difficulty or danger, without any order, to visit any such women actually receiving relief, or whom the board of guardians may subsequently decide to have been in a destitute condition, such medical officer shall be paid for his attendance and medicines by a sum of not less than ten shillings, nor more than twenty shillings, as the board of guardians may determine, regard being had to the distance from the residence of such medical officer.

Art. 13.—Provided that in any special case in which great difficulty may have occurred in the delivery, or long subsequent attendance may have been requisite, such medical officer shall receive the sum of two pounds; and if in any such case, any dispute shall arise between the board of guardians and such medical officer, such medical officer shall not receive the said sum until the poor-law commissioners shall have signified their approval of such payment on a report made by such medical officer and transmitted to them through the board of guardians of the said union.

The foregoing terms appear to be just and reasonable, and no objection will, we suppose, be taken to them.

### 4. SUBSTITUTES FOR MEDICAL OFFICERS.

Art. 14.—Every medical officer appointed, or to be appointed, in pursuance of the rules, orders, and regulations of the poor-law commissioners, shall be bound to visit and attend personally the poor persons entrusted to his care, and shall be responsible for such visits and attendances, and shall so keep any weekly return prescribed by the orders of the poor-law commissioners, as to show when the visit or attendance made or given to any pauper was made, or given by any person other than himself.

Art. 15.—Every medical officer to be hereafter appointed, shall, if practicable, within twenty-one days of the time of his appointment, name to the board of guardians some legally qualified medical practitioner to whom application for medicines or attendance may be made, in the case of his absence from home, or other hindrance to his personal attendance, and who will supply the same at the cost of such medical officer, and the name and residence of every medical practitioner so named shall be forwarded by the clerk to the guardians to each relieving officer, and to the overseers of every parish in the union.

Of course we presume that the commissioners will not enforce on the medical officer unnecessary attendance or vexatious returns. His remuneration will never be so high as to entitle the commissioners to impose on him *unnecessary* trouble. Justice, however, to them, to the poor, and to himself, demands a satisfactory one.

5. MODE OF OBTAINING MEDICAL RELIEF BY PERMANENT PAUPERS.

Art. 16.—The board of guardians shall, once in every six months, cause to be prepared a list of all such aged and infirm persons, and persons permanently sick or disabled, as may be actually receiving relief from such board of guardians, and residing within the district of each medical officer of the union, and shall from time to time furnish to each medical officer a copy of the list aforesaid.

Art. 17.—Every person whose name shall be inserted in such list, shall receive a ticket in the following form, and shall be entitled, on the exhibition of such ticket to the medical officer of his district, to obtain such advice, attendance, and medicine, as his case may require, without any order from the relieving officer, overseer, or other authority.

*Form of Ticket.*

____________________ UNION.

______________________________

Date ____________________

Good until the ________ day of ____________ 184

Name of Pauper ____________________

Residence of Pauper ____________________

Name of Medical Officer ____________________

Residence ____________________

Usual hour at which he is at home ____________

Art. 18.—Such medical officer shall, on the exhibition to him of the said ticket, and on application made on behalf of the party to whom such ticket was given, be held responsible for affording such advice, attendance, and medicines, as he may be bound to supply, in the same manner as if he had received in each case a special order from the board of guardians, or from any officer, to afford such advice, attendance, and medicine.

Art. 19. Provided always that if on complaint of any medical officer it be made to appear to the board of guardians, that any poor person who may have been furnished with a ticket in the aforesaid form shall have wilfully applied to, or sent for the medical officer on frivolous grounds, such poor person shall for the first time be admonished by the board of guardians, and on a repetition of such frivolous application, such poor person shall be deprived of his ticket, and thenceforth until the next half-yearly list be made out, shall not be empowered, except in cases of sudden and urgent necessity, to demand advice, attendance, or medicines, from such medical officer, without an order of the board of guardians, a relieving officer, or an overseer of some parish in the union.

6. CONTINUANCE IN OFFICE OF MEDICAL OFFICERS.

Art. 20.—Every medical officer, duly appointed in pursuance of the orders and regulations of the poor-law commissioners shall, unless the period for which he is appointed be expressly entered on the minutes of the guardians at the time of making such appointment, or be expressly inserted in a written contract entered into by such medical officer, and such period have been subsequently approved by the poor-law commissioners, continue in office until he may die or resign, or become legally disqualified to hold such office, or be removed therefrom by the poor-law commissioners.

We are not convinced of the expediency of such lengthened appointments. We think that it would be fairer to the medical men of a district to give them a turn in succession. We do not say that the appointment should be annual. But it might subsist for four or five years. If permanent, an interest may be generated which will make these appointments descend in families or run in narrow grooves. The son may succeed the father, or the office may be an useful part in the value of a practice sold or a partnership disposed of. Our medical brethren know best what they desire, but this is what occurs to us.

---

### IODINE FOR THE DROPSY AFTER SCARLET FEVER.*

Mr. Copeman, of Coltishall, writes in the Medical Gazette, in reference to dropsy after scarlet fever:—

" From having observed its power of strengthening the constitution, with, at the same time, a tendency to prevent inflammation and to increase absorption, I was induced to make trial of iodine. I prescribed it in the form of solution recommended by Lugol, viz.

℞. Iodin. ℈j.; Iodid. Potass. ℈ij.; Aquæ ℥vij. M. ft. Solutio Iodin. concentr.

Of this solution I ordered from 5 to 10 drops for children, and from 10 to 20 or 25 to adults, three times a day in water. In the first case in which it was used it rapidly effected a cure; in consequence of which I prescribed it in every succeeding case that presented itself, and with the same complete success."

---

### SCIRRHUS OF THE PANCREAS.*

Diseases of the pancreas are so rare, that every well-authenticated case is interesting. The following is recorded as an instance of scirrhus.

Dr. Mollan exhibited the recent parts in this case; they were taken from the body of a man aged seventy-three, who had been admitted into the Whitworth

---

* Medical Gazette, April 8, 1842. † Ibid.

Hospital, on the 28th of the preceding month. He stated, on admission, that he had been for a long time subject to pain in the stomach after eating, and that within the last six or eight months this pain had become severe and of frequent occurrence. He was considerably emaciated. He had tenderness on pressure over the epigastric region, and a tumor was perceptible, also, to the right of the umbilicus. No advantage was derived from any plan of treatment, and the man gradually sunk and died on the preceding Wednesday. About fourteen days before his death he became universally jaundiced, but had no vomiting until two or three days before death. He had been of temperate habits. On examining the stomach it was found distended with dark-coloured fluid, and its mucous coat presented marks of increased vascularity. The seat of the tumor was found to be the pancreas, the head of which appeared to be changed into a scirrhous structure; the termination of the biliary and pancreatic duct was compressed by it; the gall-bladder and hepatic ducts were greatly distended with bile of a dark-green colour; there were several tubercles in the substance of the liver, of a white colour and scirrhous structure.—*Dublin Journal of Medical Science.*

---

### Preparations of Iron.

Mr. Tyson, of Derbyshire, has published some judicious remarks on the above subject, and communicated some valuable formulæ, in the Pharmaceutical Transactions, some of which we shall introduce here.

Iron can have little effect on the human body unless dissolved by the acid of the stomach, or by some acid before swallowed.

" As this combination with acids is necessary, so physicians and chemists have diversified this combination a hundred ways, and the best preparations for the purpose of medicine, are those which are easily soluble, at the same time are rendered the least disagreeable to the stomach.

With this view, spirituous tinctures have been invented; but as some take up too small a quantity of iron, and others by long keeping deposit it again, so of course these are not without objection.

With respect to other preparations of iron, I have to observe, that the filings are often productive of inconvenience and a disturbance in the stomach, with fetid eructations, from the evolution of hydrogen gas. These effects are produced in a still greater degree, by the use of even a few grains of the sulphuret of iron; sulphuretted hydrogen being given off, which acts as a poison, and consequently this preparation is not well calculated for internal purposes.

The rubigo ferri requires to be given in large doses, on account of its insolubility, and to be long persisted in: in both cases the stomach often becomes cloyed, and sickness arises before sufficient can be taken to produce the desired effect.

The subcarbonate or precipitated carbonate of the shops, is liable to the same objection, being, as I apprehend, peroxide, or mere colcothar, and but in a slight degree soluble, which is the reason why such large quantities can be occasionally taken for a dose, whereas of the carbonate of the protoxide as formed in the mist. ferri c., or in the pil. ferri c., two very useful preparations, from five to ten grains is generally as much as can be taken for a dose, without producing considerable stimulant effects.

The sulphas ferri, occasionally produces pain in the bowels, nausea, and vomiting, especially if taken in improper doses, or long-continued.

The most valuable preparations of iron are those which have the deutoxide for their base, as in the mineral waters, and in the formula I am about to give you:—

LIQUOR OXYSULPHATIS FERRI.

℞. Ferri sulphat. ʒij. (or ʒiij.)

Acidi Nitrici ʒiij.

Aquæ Dist. ℥iss.

Tere diligentèr per horæ quadrantem acidum nitricum ferro vitriolato, dein sensim addendo aquam, per chartam cola, et fiant guttæ, é quibus capiat æger gtt. v—xij. bis in dies ex infuso quassiæ vel aquâ.

This form, I believe, was invented by Sylvester, about forty years ago, and has ever since that time been in constant use among the practitioners of Derbyshire. I wonder it has not been inserted into the Pharmacopœia, as it is by far the best and most powerful of all the preparations of iron. The oxygen of the nitric acid uniting with the sulphate of iron, forms a persulphate; at the same time the iron is converted into red oxide. As a medicine it far surpasses the tinc. ferri mur., and it never precipitates the oxide of iron. It is one of the most valuable restoratives in the debility and torpor of the liver, which remains after the successful treatment of hepatitis. Patients do not well bear above ten or twelve drops to a dose; and when given with small doses of sulphas magnesiæ, &c., it equals the purgative mineral waters. I think it will be found to be an antidote to prussic acid, as it instantly combines with it."

---

### COUNTER-IRRITANTS.

The following formulæ have been communicated by Dr. Turnbull to the Editors of the Pharmaceutical Transactions.

*Tinctura Capsici Concentrati.*

℞. Capsici Baccarum, ℥iv.
Spiritus Vini Rect. ℥xij.

Macera per dies septem et cola. (It may also be made with advantage by displacement).

This concentrated tincture is used as an external application, and is found to be a powerful rubefacient and counter-irritant, for which purpose the ordinary tincture of capsicum is not sufficiently potent.

VERATRIA, dissolved in this tincture, acquires increased activity; the capsicum apparently facilitating its absorption into the skin. Four grains of veratria, dissolved in an ounce of the concentrated tincture of capsicum, will be found as powerful in its effect as twelve or fifteen grains dissolved in alcohol.

*Pulvis Aluminis et Capsici.*

℞. Aluminis Sulphatis, partes tres.
Tinct. Capsici concentrati, partem unam.

Misce et sicca.

A very small quantity of this powder, applied to the tonsils, is found more efficacious, in some cases, than an alum and capsicum gargle.

*Unguentum Ipecacuanhæ.*

℞. Pulveris Ipecacuanhæ, ʒij.

Olei Olivæ, ʒij.

Adipis, ℥ss. M. ft. unguentum.

*Unguentum Emetinæ.*

℞. Emetinæ, g. xv.
Sp. Vini. Rect. q. s.
Adipis, ℥ss. M. ft. unguentum.

Dr. Turnbull states, that he has found this ointment particularly efficacious as a rubefacient in pulmonary and rheumatic affections, producing little or no pain or inconvenience to the patient."

---

### Summary of the Mortality Tables of London for the Year 1841.

The following brief summary is contained in the number of our northern contemporary,* for May 1842. It is abstracted, we presume, from the Registrar General's Report, which we have already directed attention to.

The total number of deaths was 45,284. Of these 22,995 were males, 22,288 females. 20,780 were under 15, and 24,433 above 15 years of age.

In the tables, the area is divided into five districts, and the rate of mortality is found to differ in each, according to the local situation, and the circumstances and occupations of the inhabitants. Thus, in the western district, embracing Westminster, St. James, Kensington, &c., it is 2,202 per cent.; in the northern, embracing St. Mary-le-bone, St. Pancras, Islington, and Hackney, it is 2,267; in the central, or what is properly called the city, it is 2,505; in the southern, embracing Southwark, Greenwich, Camberwell, &c., it is 2,539; and in the eastern, embracing Shoreditch, Whitechapel, Bethnal Green, &c., it is 2,558, shewing a difference of .356 per cent. between the western, which is the wealthiest and best aired district, and the eastern, which includes the poorest and most low-lying parts of London, along the banks of the Thames. The average mortality over the whole, is 2,429 per cent. The central district is entirely urban, the eastern nearly so, while all the others comprehend a considerable extent of surrounding country.

The tables exhibit the number of deaths which occurred in each week, and their causes. The diseases are classed as follows: I. Epidemic, endemic, and contagious. II. Spasmodic, embracing, 1st, diseases of the nervous system; 2d, of the respiratory organs; 3d, of the organs of circulation; 4th, of the digestive organs; 5th, of the urinary organs; 6th, of the organs of generation; 7th, of the organs of locomotion; 8th, of the integumentary system; 9th, of uncertain seat. III. Deaths from old age; and IV. Deaths by violence.

The mortality from the exanthemata and typhus, amounted to 3,840, of which 1053 were from small-pox, and 1,151 from typhus—certainly a very small number, considering the extent and circumstances of the population. The deaths from consumption were 7,326, or about 16·17 per cent. of the whole, being nearly the same proportion as in the rest of the kingdom. The mortality from the other principal diseases, was as follows: Hooping-cough, 2,278; hydrocephalus, 1,739; convulsions, 2,778; pneumonia, 3,668; asthma, 1,351; dropsy, 1,720; debility, 1,114; old age, 3,373. The violent deaths amounted to 1,148. The mortality in the first quarter, amounting to 13,713, far exceeded that in the others, which differed little in this respect, being 10,404, 10,406, 10,761, respectively. The month in which the greatest number of deaths occurred, was January—4,687; that in which the least, July—3,050, showing a difference of 50 per cent. The higher mortality of the first quarter holds good with respect to all the great classes of disease, whether epidemic, endemic, or spasmodic, with the single exception of diseases of the digestive organs, from which the greatest number of deaths occurred in the autumnal quarter, owing to the prevalence of gastritis and enteritis at that season. In the case of individual diseases, there are some exceptions to this remark. Thus, while the mortality from

---

* London and Edinburgh Monthly Journal of Medical Science.

pneumonia, bonchitis, and asthma, was far higher during the first quarter, that from consumption was highest during the second and third quarters, and from croup in the fourth. The number of sudden deaths, from unknown causes, was greatest in the first quarter, and the deaths in child-birth were twice as numerous in the first, as in the second and third quarters.

Of the four years, during which the deaths have been registered, viz. from 1838 to 1842, the most fatal, 1838, was also the coldest, while in 1841, the least fatal, the temperature was higher and more rain fell than in any of the other three.

---

### OX-GALL.

Dr. Clay, of Manchester, has published some valuable observations on the internal exhibition of inspissated ox-gall, in our rising and rapidly improving contemporary, the MEDICAL TIMES, which we shall take the liberty of transposing to our pages. Our readers are well aware that we have often alluded to this subject, and published some cases illustrative of the powers of this substance in certain diseases, especially jaundice, dyspepsia, &c.

" *On the Exhibition of Inspissated Ox-gall in various Diseases. By Charles Clay, Member of the Royal College of Physicians, London, &c. &c. Lecturer on Medical Jurisprudence, &c. Manchester.*

" I have had my attention for some time directed to this somewhat novel article of the materia medica, with a view to ascertain its powers as a medical agent, to what cases it appeared most applicable, and the best method of administering it. Gall of animals is by no means a new remedy, for I find its use spoken of by Boerhaave, and since his time by various writers ; full justice, however, has not been done to its merits, it only having been tried in isolated cases, no one taking the trouble to test its powers by frequent experiments either on the same or different diseases. Boerhaave relates, 'that he has cured pale ricketty children by pills made of the galls of the eel and the pike; that the medicine operated powerfully by urine; and that by its use the belly, before swelled, subsided surprisingly.' Lewis, in his materia medica, says, ' in want of appetite and other complaints proceeding from a deficiency of bile in the first passages, this animal bitter may probably be of more service than those of the vegetable kingdom usually directed in such intentions.' As an external application, ox-gall, combined with the camphoretted spirit of wine, has been often spoken of in rheumatism, sprains, and bruises. From experiments made upon gall by Cartheuser, Baglivi, and others, it was found to be very soluble in water, sparingly acted upon by rectified spirits, rendering oily, unctuous, or resinous substances, miscible with water; it *has the peculiar property of preserving milk from coagulating, or turning sour, or when coagulated immediately dissolves it again ;* this last property deserves particular notice, and to which I intend to allude afterwards. So far as experiments have been instituted on the gall of animals, there does not appear to be any great difference in its composition, and as the gall of the ox is much easier to be obtained in large quantities, I have selected it as the object of the following experiments :—Dr. Peacock, of Darlington, remarked (Lancet, vol. 1, 1836-7, page 398), that he had observed in a case of scirrhous or cancerous ulcer of the breast, that when the system exhibited an accumulation of bile, the pains accompanying such diseases (cancerous) were very greatly alleviated. In the case from which he drew this inference, during the progress of the disease, the patient was frequently attacked by the symptoms of jaundice, and invariably when the white motions and yellow skin appeared, there was almost entire relief from pain. Although Dr. Peacock exhibited gall in other cases, he does not give any decided opinion upon it, but augurs favourably and wishes its being further tested by others. Having some cases analogous, I determined on giving it a trial in such affections, before I proceeded to its trial in other diseases, towards the cure of which I fancied *its powers more applicable.*

Case 1.—Anne H——ly, æt. 56, had a cancerous ulcer that had destroyed the greater portion of the nostrils, accompanied with the most violent lancinating pains, over which neither local applications nor internal medicines (except for a very brief space of time) had any control; the case had been long under treatment without improvement, and as a forlorn hope, I ordered the following:—

℞. Fel. bov. inspiss., ʒij.;
Ol. carui., m. x.;
Magnes. carbonatis q. s. ut fiat massa. M.
Div. in pilulas xxxvi., capiat ij ter in die.

In order that the effect might not be combined with any other object, no local application beyond clean washing was recommended. After taking the pills for only one day great relief from pain was experienced: on the fourth day no trace of pain remained; for three weeks the sore improved in appearance, without pain; slight pains again occurred. The dose was ordered to be repeated four times in the day. The pains again subsided, and all progressed well for two weeks longer, when the pains again occurred. Pills ordered, two repeated six times in twenty-four hours, after which the pains slightly abated, but soon recurred again. Eight weeks after the commencement the sore began to assume its old character, and the pain became violent; she soon after left the dispensary, and I lost sight of the ultimate result. Before giving the gall in this case, the *motions were particularly white, and the bowels very much constipated, with acid risings from the stomach,* all of which vanished whilst under the influence of this medicine.

Case 2.—Hugh C—lt—on, æt. 64, of Manchester, had a scirrhous ulcer of the right mamma produced by an injury. Some years before a large fungus had been removed from the part by myself, but the ulcer remained without any disposition to close, sharp shooting pains extending to the right armpit, with an extensive and disagreeable discharge; appetite generally very deficient; bowels very torpid, and when moved by purgatives became immediately torpid again; *his motions were particularly white.* The treatment of this case had been various; some relief from pain, and a better disposition to heal, with less discharge from the sore, was observed (for a short time only) by the application of the pulveris ferri carbonatis in the form of ointment, and the occasional exhibition of the pil. hydrargyri c. opio internally. On giving the inspissated ox-gall to the extent of twenty grains in the day, according to the formula in case 1, a decided relief from pain took place, the sore put on a healthy appearance, his nights (before restless) were comfortable, and the discharge from the sore of a less offensive character. About a fortnight after taking the inspissated gall the pains returned; I increased the quantity to thirty grains in the day, when he again experienced the same relief; he has now taken it nearly three months, occasionally omitting it for a few days; the sore is very nearly healed, his general health very much improved, and his bowels very regular.

Case 3.—Mrs. J——son, Ancoats, Manchester, æt. 70, with an extensive cancerous destruction of the whole left labium pudendi, extending rapidly to the mons veneris and the glands of the left groin; no local application or internal medicine gave the least relief, except large doses of the mur. morphiæ, and then of the most temporary character, seldom producing more than a single hour's sleep at a time. In a case so utterly hopeless, I had not the least expectation of giving more relief (if as much as had already been obtained by the mur. morphiæ). The digestive powers were almost defunct, the stomach rejecting the greater portion of food, and what was retained produced violent pain; the bowels, which were extremely torpid, seldom evacuated, *and then the motions were of a chalky*

*nature*. I ordered her thirty grains of the inspissated gall per diem. Calling upon her the day after she commenced taking it, I found she had enjoyed more rest, and had been more free from pain than for many months past. After taking it six weeks the pains were becoming violent again, and the dose was increased to forty grains per diem, which dose she now takes. It gives more relief than any other remedy, in consequence of which she sleeps better, her bowels are more regular, and the ulcerous discharge less offensive; but the work of destruction still progresses rapidly, and evidently to a fatal termination.

*Observations on the above.*—It is evident, then, that the inspissated gall is but a palliative in cases where scirrhous or cancerous diseases have assumed a malignant form; but even under such circumstances it is a valuable acquisition to the means of giving relief, though temporary; and it is probable, that if exhibited in such diseases at a much earlier period, the relief might be more certain—nay, a cure might possibly be effected. Dr. Peacock mentions the decided character of the motions *(being white)*; the same appearances with obstinate constipation and deranged digestion were observed in each of the above cases. On turning over numerous authorities, I find the same sluggish tendency of the bowels, with indigestion, generally mentioned by all, which, in my opinion, goes far to prove that all scirrhous affections arise from a want of sufficient secretion of healthy bile; or, in other words, that where there is a deficiency of bilious secretion, there is a tendency to disease, and ultimate lesion of continuity in some structures facilitated by previous injury—thus, the granular structure is often the seat of such affections; hence the mammæ of females are very liable. If this theory be a correct one, no better means can reasonably present itself for relief than by supplying the system with that of which it has hitherto been deficient. The effect produced in the above cases strengthens my opinion of its usefulness in cases more immediately connected with the alimentary canal, which I shall now proceed to illustrate.

CASE 4.—I was myself labouring under dyspepsia, almost every kind of food became acid soon after being taken; I had violent headaches, constant pain in the epigastric region, and bowels very much constipated, many times three or four days without a motion; occasional relief, but of a very temporary nature, was obtained by the pilulæ hydrargyri. Having been subject to these symptoms, more or less, for seven or eight years, but which have been often very severe during the last three or four years. Purgative medicines always produced great irritation and uneasiness for some time after their exhibition. Under these circumstances I took two four-grain pills of the inspissated ox-gall, not having had any motion for nearly four days; the pills were taken at four o'clock in the afternoon, and at seven, without even the slightest sensation of pain, or the common feelings arising from having taken purgatives, I had a free and copious motion, the excrementitious mass being in a pulpy form, and perfectly free from the indurated character I had been so long accustomed to. I repeated the dose next day with similar results. I experienced not even the slightest feeling of uneasiness; indeed, had I not known the fact, I should not have supposed that I had taken medicine of any kind. The acidity immediately left my stomach, and when under its influence the pains in my head and stomach were removed, and my bowels are now quite regular. From taking occasional doses, of course its effects are not sufficiently tested; but I have experienced more relief, with less unpleasantness, than from any other of the many means I have ever resorted to; in fact, its value in dyspeptic stomachs is incalculable.

CASE 5.—Mrs. W—g, the wife of a painter, thirty-eight years of age, and mother of five children, had been subject to a very constipated habit from a very early period of her life, not unfrequently the space between evacuations extending twelve or fourteen days, and very commonly to ten days, whether pregnant or not, the same tendency to constipation existed: she had sought advice from medical men of the highest repute and experience, but at most only received temporary relief, her bowels almost immediately relapsed into their accustomed state, in spite of the best efforts to the contrary. In the endeavour to procure motions, she had frequently taken from ten to twelve ounces of the ol. ricini, and twenty or thirty drastic purgative pills before an operation could be procured, and then with great pain in the bowels, vomiting, &c., only parting with small balls of indurated fæces. Before giving the inspissated gall, I thought it advisable to test the obstinacy of the bowels by other means, and therefore ordered the following mixture:—

℞. Alb. ovi. f. ℥j.
Olei ricini f. ℥ij.
Olei menthæ, pip.
Olei crotoni, āā. m. iij.
Aqua puræ f. ℥vj. M.

Sumat æger cochlearia duo magna quâque tertiâ horâ.

After persevering two days with this, only one small motion was passed, and that with violent vomiting, and almost intolerable pain, the fæces having the character of hard balls. Waiting two or three days for the excitement to subside I then ordered the inspissated gall, according to the formula in case 1, giving two four-grain pills three times a day, or twenty-four grains in the day; in little more than six hours from the first dose, and before taking the third, she had a very copious motion, without the slightest degree of sickness or pain, except for a few moments, caused by the hardened fæces passing the sphincter ani; on the second day a second, and a third motion, as easy as the first, were passed, when I extended the intervals between the doses of the pills to six hours, viz., sixteen grains per diem: from this time her bowels were perfectly regular, and without the slightest uneasiness. Three weeks from the commencement of taking the inspissated gall, she omitted the pills entirely for three days, when the bowels were disposed to assume their former state; resuming the pills once in the day she became quite regular. It is now eight months since the first trial with the gall, and having left off the pills entirely for the last two months, she is quite regular in her bowels. The effects of the inspissated gall in this case were decided, and highly satisfactory. A more obstinate case, showing a more favourable result, could scarcely be adduced.

Case 6.—Mrs. Ll—d, also a painter's wife, 28 years of age, accustomed to weighing out white lead paint to the workmen for five or six years past, during which time she has been subject to obstinate constipation of the bowels, averaging one movement in seven days. It was with great difficulty that motions were procured oftener by medicines of any kind, although she had tried numerous applications, and then with great pain and sickness; the motions when passed were like hardened white marl, evidently characteristic of a deficiency in the bilious secretion. I may here also state the same appearance of the evacuations accompanied those of case 5. I ordered twenty-four grains of the inspissated gall per diem; the very day after, her bowels were freely and copiously moved, and have continued perfectly so since, after taking the gall for a fortnight; she reduced the doses from three to one per diem; that is, eight grains every morning for three weeks longer. It is now only taken occasionally, the bowels are quite regular, and as in the cases before stated, not the slightest feeling of pain or sickness occurred during its exhibition, the parties taking it experiencing no symptoms of being under the influence of even the simplest laxative medicines.

Case 7.—John D—tt—n, a working painter, æt. 30, in the habit of sleeping in the workshop for the better security of his master's property; had been subject to constipation of the bowels since he was an apprentice to the trade (about fifteen years); motions only occurring about every seventh or eighth day, and then of a chalky indurated character; his countenance was sallow, appetite bad, and he had frequent attacks of the colica pictonum. I commenced treatment in this case by giving the oleaginous mixture as in case 5, but with little effect, and that at the expense of great pain and sickness; the motions procured were hard, knotty, and very small in quantity. I then ordered thirty grains of the inspissated gall per diem; on the second time of his taking it, the bowels were copiously moved without an unpleasant symptom; he has taken it about a fortnight, and the bowels are now quite regular, and the motions of proper consistence; he expresses himself more comfortable in his feelings, with a better appetite for food than he has experienced for many years.

In addition to these cases, I may mention I have given it in the common constipation attendant on pregnancy with the best effects.

*Observations on cases* 4, 5, 6, 7, *&c.*—I cannot reflect on the effects of the inspissated gall in the cases 4, 5, 6, 7, &c., without great satisfaction; since it proves, as I anticipated, a most valuable adjunct to our means of improving the condition, and correcting the mischiefs, of the alimentary canal; and as the diseases connected with it are evidently either entirely cured or very much ameliorated by its exhibition, I think there can be no doubt that where obstinate constipation exists, whether it be the cause, or the consequence of the present abnormal state of the system, it arises either from a vitiated property of the bilious secretion, or a deficiency as to the quantity secreted; *if so*, the substitution of inspissated healthy bile must improve the condition, and the quality of what is already in the alimentary canal, producing also a proper effect on the fæcal mass; *if there be a mere deficiency in quantity*, the introduction of an additional supply must better answer the intentions of nature in performing her excretory operations. If I take the whole range of cases hitherto introduced as illustrative in this essay, *deficiency in quality or quantity of bilious secretion is the prominent and prevailing accompaniment.* If I were asked how the inspissated gall acts so as to procure a more soluble state of the fæcal mass, in such cases as cited in Nos. 5, 6, and 7, I should say distinctly, neither as a laxative, purgative, nor drastic, all such producing, to a greater or less extent, a stimulus to the intestinal coats, exciting them to propel their contents and to excite an extra secretion from the exhalents (the latter action, however, in my mind, is rather questionable); such is the generally allowed operation of the various degrees of cathartic medicines, and the common consequence arising from taking such is nausea, sickness, griping pains, &c., more or less, according to the character and dose of the medicine employed. Inspissated gall, on the contrary, produces not the slightest pain or sickness, and yet a motion can with equal (or greater certainty) be relied upon, and that in a form most easy and natural for propulsion. It is evident its action is not as a cathartic, but as a direct solvent to the accumulated hardened fæcal mass, *the consequence of deficiency of quality or quantity of bile in the alimentary canal;* as such, its effects may be produced without pain or uneasiness, which would not be the case if its action was on the principle of cathartics. I shall, in my next communication, show the effects of inspissated gall in diseased livers, dropsies, and in that numerous class of diseases arising from acidity in the first passages of children, with some experiments on the bile and fæcal matter.

In continuing my practical observations on this question, I would remark that the constipation attendant on working in frequent contact with white lead, would lead to the conclusion that it had a direct tendency to arrest the secretion of bile, not only as to quantity but quality, so as materially to impede the healthy process of digestion and excretion of fæcal matter. I have never yet seen a case of painters' constipation without the white motions indicative of this deficiency. The cases 6 and 7 are examples of this. In respect to case 5, though frequently in contact with white lead, yet the habitual constipation had been observed from infancy, and therefore could not be directly attributed to the effects of lead, as the same deficiency of bile had been observed in the motions from her earliest recollections. The case was one of the most obstinate character, and no doubt rendered worse by the husband's occupation, in which she assisted. It appears to me contrary to common sense to attempt to evacuate the intestines of fæcal matter by drastic purgatives (except by those of an oleaginous nature), the inner coat of the intestines being almost destitute of mucous secretion, and the fæces hardened by the absence of bile; an attempt to move the bowels by drastics under such circumstances must endanger inflammation. But if (as I shall afterwards prove) ox-gall directly dissolves the hardened fæces, and by that action alone renders them easier of propulsion, by the addition of bile I directly overcome the constipation, and by charging the system with an extra quantity of healthy bilious secretion I prevent its recurrence, giving time for improving the secretory powers of the liver by other remedial measures. In cases of constipation produced by the exhibition of opium, it is always remarked that the motions are clay coloured, evidently proving that less bile is secreted than ought to be, opium having a more decided effect on the secretion of the liver than that of any other organ in the body; hence (the bilious secretion being checked, or materially diminished by the presence of opium) follows that obstinate and frequently mischievous *constipation* so often met with in practice, and more particularly in children, who are so often crammed with sleeping nostrums. When I have found it necessary to employ opium in the treatment of disease where I wished to avoid its peculiar tendency to constipate the bowels, I have always succeeded by combining it with the inspissated gall, which in no way impedes the desired action of the opium, whilst it is an effectual preventive to its confining the bowels.

Case 8.—William Macartney, æt. 56, had a constantly irritating dry asthmatic cough, which only gave way to the pil. scillæ comp. c̄ opio at bed-time in large doses; but in consequence of their tendency to confine the bowels, he was obliged to omit them, to the great sacrifice of his rest and comfort, and take purgatives. I ordered him eight grains of inspissated gall every morning, and the pil. scillæ comp. c̄ opio at bed-time; from the commencement of his taking the gall pills the bowels became quite regular, and have remained so ever since: thus the gall perfectly counteracts the constipating effects of the opium, whilst its sedative effects appear in no way diminished.

In addition to the cases already enumerated, I might add many others of dyspepsia in treatment and result so nearly like my own (case 4), that a repetition of particulars will, therefore, be unnecessary; suffice it to say, they have all been effectually relieved by the same means, their stomachs and bowels assuming a regularity quite new to them, without any apparent effort, or causing the least uneasy symptom.

The following cases are in connexion with extensive organic disease of the liver, in which the inspissated gall was of considerable benefit in preventing the strong tendency to constipation which existed.

Case 9.—Mrs. P—t—ton, æt. 65, for a long time affected with a scirrhous affection of the liver, with great enlargement of that organ; the bowels often very torpid, motions chalky, and when so, suffered severely from pain, loss of sleep, &c. Independent of other treatment, I gave the inspissated gall to the extent of eight grains night and morning, and it was very remarkable how much more regular the bowels immediately became under its influence; and though the organic disease was too extensive for any permanent relief, yet the ease she experienced from her bowels being more regular, was a sufficient inducement for her to prefer taking the inspissated gall pills to any other remedy previously tried, which indeed had scarcely any effect at all upon her.

Case 10.—Anne C—l—n, æt. 30, had chronic inflammation of the liver, accompanied with obstinate constipation, whitish motions, &c; after leeching, blistering, hydrarg. chlorid. &c., the obstinacy of the bowels still existed. I then ordered eight grains of the inspissated gall every night and morning. Immediately a great change manifested itself in the appearance of the motions, and perfect regularity was established, with a slight increase of urinary secretion (*the only adult case* in which I had observed any distinct action on the kidneys). The case is now quite well, not the slightest trace of the liver affection remaining. In addition to cases of this description, I might add the opinion of Dr. S. Johnson of this remedy, as recorded in the Lancet, vol. 1, 1840-1, page 447, where he states from fifteen to thirty grains per day had been administered by him with the best possible effect in bad cases of jaundice; it was indeed this statement that first induced me to test its merits at greater length, in which I have not been disappointed. In respect to the action of ox-gall on the kidneys in adults, I very much question its utility, as I have not been able to perceive any increase of urinary secretion in the foregoing instances except in case 9, and that very limited, and which might with equal propriety be attributed to any of the other means employed, I therefore do not think it promises much in cases of dropsy. The high authority, however, of Boerhaave, as quoted at the commencement of these observations as to its effects on children, increasing the secretion of urine, cannot be passed over. I have not given it to marasmoid children to that extent to speak decidedly; but in the cases in which I have had an opportunity of exhibiting it, I have observed a decided and marked improvement of the system—the tympanitic state of the belly reduced—appetite improved—motions of a bilious character—*and an increase of urine.* In all cases of marasmus, whether of children or in the atrophy of adults, I have in ox-gall a valuable remedy. In acidity of the stomach, &c. of children, it is of most decided, effectual, and immediate relief. The curdled vomitings, green motions, abdominal gripings, and restlessness immediately disappear, and a better state of general health is substituted; in all such cases there was a decided action on the kidneys, increasing the secretion. On looking at its effects upon children as just stated, particularly whilst at the breast, living almost entirely on milk, the result is not different to what we might suppose when considering the experiments of Baglivi, Lewis, &c. *'That it prevents milk from turning sour, and dissolves it when in a state of coagulation;' an antacid preparation is indicated, which is one of the peculiar properties of this remedy.* To show its direct effect upon hardened fæces, a child of sixteen months old passed a very hard motion with very great difficulty, not having had one for three days. I poured a solution of ox-gall over it in a vessel, immediately its chalky appearance was changed to a more healthy bilious colour, and reduced to a pulpy mass in half an hour; from this fact, I will suppose a case, (one which has frequently occurred in my practice,) an adult with hardened fæces in the rectum, almost, if not impossible, to pass without assistance; under such circumstances, what could afford a better prospect of relief than two or three ounces of recent gall, diluted with as much water, used as an injection. It is needless to observe I would pledge myself as to the result, viz., an immediate softening of the mass facilitating its propulsion.

So far as these experiments have progressed, the use of ox-gall in some diseases is of the most satisfactory character, presenting us with an excellent and peculiarly effective corrective for the many and various derangements of the alimentary canal, unlike many of our best medicines, inasmuch as in whatever cases it is given, if no benefit results, no harm is ever experienced from it. Its action on the system is not as a purgative, but as a mere solvent of the *material* contained within the intestinal canal, producing no excitement to propel, but by liquifying the mass, facilitating its excretion. It is also a tonic—and in children to a moderate extent, a diuretic—but less so in an adult. It appears to have a a peculiar and specific action on all that variety of diseases connected with derangements of the digestive organs, and from the proofs I have advanced, I believe it worthy of extensive trial. The preparation I have been in the habit of giving, is simply the recent gall of the ox slowly evaporated to the consistence of an extract, and afterwards made into pills, as in the formulæ already given; but if it is sufficiently firm, I prefer the simple extract made into pills without any addition; and if the gall be *recent*, it has very little smell, but an intensely bitter taste. The gall-bladder of a moderate sized ox will afford as much extract as will make one hundred four grain pills, and is an article both cheap and easy to procure. Trusting it may be further tested by others, I leave it to the profession, confidently recommending it to their notice.

The value of opium as a remedy in disease is acknowledged by all practitioners, and it would be much more generally given if it were not for the constipation resulting from its application, by the general check it is supposed to give to the operation of the secreting organs. How this is accomplished is not so easily explained; physiologists admitting, that though digestion may progress during sleep, secretion is but very slowly carried on. The action of opium, then, may not be direct on the secretory organs, but by producing artificial sleep, *through that sleep* produce the check to the secretions, generally attributed to the direct effect of the opium itself.

Perspiration, however, may be adduced as a contradiction to this, but it may with equal propriety (as it has been frequently) be denied that perspiration *is a secretion*; this (perhaps properly called) exudation being most certainly and frequently produced by sleep, it also follows that the perspiration attributed to various preparations of opium, 'pulv. Doveri, &c.' may not be the direct effect of the preparation used, but the consequence of the sleep produced by it. Dr. Holland, in his 'Notes and Reflections,' observes, 'the fear of confining the bowels, and checking the secretions, constantly present to the mind of the practioner, prevents the adequate use of a medicine, having the power of mitigating pain, relieving spasm, procuring sleep, and producing perspiration, &c.'

In whatever view the action of opium is considered, it must be acknowledged that if its action *can* be secured without the disadvantages generally attributed to it, its value as a remedial agent must be very considerably increased, and the frequent objection to its use be done away with. That such *can* be *positively* accomplished by the combination of ox-gall with it, the cases here given fully prove. And supposing the secretion of bile in the system to be deficient in quality, or quantity, originating disease from whatever cause, there cannot remain a doubt but that an artificial supply of bile must be attended with the best possible results; that *substitute* acting in the full capacity of the original secretion, until such secretion be either amended or restored. Ancient pathologists, particularly of the Hippocratic school, attributed considerable importance to bile: and perhaps moderns have too much neglected the results arising from its redundancy or deficiency in the animal system. What I have hitherto advanced has been chiefly to illustrate the deficiency of bile in the alimentary canal; but before I conclude this subject, it may be necessary to observe very briefly, that diseases not unfrequently arise from the bilious secretion being too great in quantity, and what necessarily follows, depreciated in quality, hence those obstinate and long-continued diarrhœas, and cases of jaundice, *accompanied with purging*, (*which are very distinct* from the common run of cases of jaundice,) being the consequence of mechanical obstruction in the biliary ducts, always accompanied with constipation, little or no bile passing along the alimentary canal. But I have frequently observed cases of jaundice, and more in infancy than adult ages, where no such obstruction existed, and where motions evidently indicated a full sufficiency of bilious secretion. Jaundice arising from obstruction in the biliary ducts *may*, after the mechanical obstruction has been removed by emetics, &c., be much benefitted by the exhibition of gall internally, which not only assists the bowels in passing off the excrementitious mass, but improves the secretion of bile so as to prevent the formation of gall-stones in future. But those cases accompanied with lax motions, as well as long standing cases of obstinate diarrhœa, would rather be injured than benefitted by it. The only resource in such is to lessen the bilious secretion, and I know of no one object more efficient in checking that secretion, and exciting perspiration, than small and frequent doses of crude opium, with warm clothing. If once the exudation by the skin becomes apparent, in such cases it may very certainly be predicated that the secretion of bile is lessened, and the case progressing towards a cure. I am convinced there exists a remarkable sympathy between the secretion of the liver and the exudation by the skin, either of which being lessened increases the other, and vice versa.

The case of diarrhœa reported by Dr. Clendinning in the 'Medical Times' since the first part of these observations were written, I should rather attribute to a too free secretion of vitiated bile, than to the locality in which it occurred; and the warm clothing he so judiciously advised, by promoting perspiration, lessened the bilious secretion, and so checked the diarrhœa. I have witnessed many similar cases with similar results. I think there is much reason for supposing that the bilious vomitings, motions, &c. as observed in Asiatic cholera, are in consequence of a too redundant secretion of vitiated bile, further confirmed by the sympathetic state of the skin, dry, cold, and shrivelled; in such cases inspissated gall offers itself with every probability of improving the condition of the system when combined with opium. I only speak on supposition, as I have not had an opportunity of testing its powers in that dreadful scourge. Another remarkable disease falls within the range of supposition—viz. diabetes, now pretty generally allowed to be, in the first stage, an attack of the stomach, proved by the acidities, constipation, &c. present; here again is perceived the strong connexion between the organs of digestion and the skin, being almost, if not perfectly, free from exudation. These symptoms are so strictly analogous to those in which inspissated gall has been so decidedly effective, that I would not hesitate to anticipate great advantage from it, if not a direct cure. I do not wish to be misunderstood. I do not mean this to apply to the secondary stage of diabetes, where the urinary organs are so extensively deranged; when that occurs, I have had great success in the exhibition of the tinct. ferri. sesquichloridi (see cases reported in Lancet, vol. i. 1840-1, p. 84). I have not, however, had any cases of diabetes since I began to apply the inspissated gall to practice to test its merits upon, but I think it capable of correcting the evil, and certainly worth trial, since so little satisfaction has been derived from any means hitherto suggested, any new plan is justifiable."

---

### DORTON CHALYBEATE.

There are but few, either in or out of the profession, who know anything of a strong chalybeate (Dorton) situated 12 miles from Oxford, and six from Thame, which has no small local reputation in the neighbourhood for the marvellous, not to say miraculous cures which it has effected. The water rises clear, and has a strong inky taste, and a peculiar though not unpleasant odour. When exposed to the sun and air, a slight pellicle appears on the sides of the glass and surface of the water. Soon after this it lets fall a light brown sediment, which may be re-dissolved by the addition of a few drops of sulphuric acid. The well exhales a rather strong sulphureous odour; but unless the water is taken at the fount or bottled immediately, this odour is soon lost. When first poured into a glass it exhibits a momentary sparkling appearance, and when swallowed in that state produces a refreshing and exhilirating effect. When mixed with powdered galls or strong tea it becomes quite inky—or rather ink. The following are the leading contents of a pint of this Spa, as ascertained by Professoı Brande.

| | |
|---|---|
| Traces of carbonic acid | |
| Sulphate of lime | 11½ grs. |
| Muriate of soda | 1½ |
| Sulphate of alumine | 2 |
| Sulphate of iron | 10 |
| | 25 |

This Spring contains infinitely more iron than Tunbridge Wells, and one-third as much as the celebrated styptic water of Sandrock in the Isle of Wight. From the above analysis it is evident that people of full or plethoric habit, or with costive bowels, should be extremely cautious how they use the Dorton Chalybeate. In almost all cases, it is advisable to take the Spa diluted with an equal quantity of warm water. Mr. Knight, who has written on this Spa, informs us that, in cases of dyspepsia, attended with loss of appetite, flatulence, tremors, nocturnal watchings, low spirits, &c. a patient trial of this chalybeate has often produced permanent relief. Patients of this description should take two wine-glassfuls an hour before breakfast—and the same quantity an hour before dinner. In a few days the dose may be increased to a moderate tumbler-full. A brisk walk, immediately after taking the water, will promote its good effects. In this, as in all other complaints, the bowels should be well cleared before commencing the chalybeate, and carefully attended to during the course.

It has acquired considerable reputation in chorea—leucorrhœa—menorrhagia from debility—chlorosis—and in all relaxed and cachectic states of the constitution. In these conditions, the water is not only taken internally, but used as a tepid bath also, as an auxiliary. In scrofulous sores, and a variety of cutaneous complaints, especially psoriasis and lepra, the waters of Dorton, inwardly and outwardly, have acquired considerable fame. In herpes and tinea, the same may be said.

The Spa is delightfully situated, a mile from the romantic village of Brill.

"A lovely village seated on a hill,
Where Nature wears a most bewitching mein,
Where beauteous scenes, enchanting prospects fill
The enraptured soul, with extacy supreme."

Mr. Knight has published some remarkable cases that have been cured by the Dorton Spa. We shall only notice one. A married female was severely afflicted with lepra pervading the whole body, with the exception of the face. Dr. Ferguson, of Windsor, had tried various remedies, including arsenic, without relief. The thick scales on her legs, when cracked, discharged through the fissures considerable quantities of blood, as well as humour. The waters, both internally and externally, were employed vigorously. The desquamations daily were astonishing, the incrustations resembling pieces of leather. The baths were taken at 110° of Fahr. and kept up for half an hour, every second or third day. The skin rapidly attained its wonted appearance, and ultimate recovery took place. Mr. Knight used saline medicines in aid of the waters. We think the medical gentlemen in the neighbourhood should experiment on this powerfully tonic Spa.

---

### Some Cases of Sudden Death from Hæmorrhage of the Lungs in Children. By Cathcart Lees, M.B.*

*Case* 1.—A delicate boy, æt. 6, of lymphatic temperament, had been labouring under tubercular phthisis for some time, when he was suddenly attacked on the 1st of March with profuse hæmoptysis, which subsided under treatment; this returned on the 4th, and he expired while coughing up large clots of blood.

On examining the chest, a large quantity of sero-sanguineous fluid was found nearly filling the cavity of the left pleura, and in the inferior lobe of the left lung was a cavity, of the size of a large nut, filled with coagulated blood; the parenchyma of the lung forming the walls of the cavity was softened, irregular, and of a dark colour, not lined by any membrane, nor were there any bands crossing it, but on passing a probe through the division of the pulmonary

* Dublin Journal, May 1842.

artery leading towards the cavity, a large branch was found to open into by a wide aperture, with ragged edges, and which had evidently furnished the hæmorrhage.

The substance of the lung around was softened, and presented a gangrenous odour. A large bronchial tube opened into the cavity, which with the trachea was filled with coagulated blood. The apex of the lung was studded with miliary tubercles.

*Case* 2.—William Hall, æt. 3, of scrofulous diathesis, had been labouring under tubercular phthisis ; he was attacked suddenly with profuse hæmorrhage, which ceased for a time, and recurred fatally on the third day.

A tubercular cavity, of the size of a pigeon's egg, was found in the posterior part of the apex of the left lung, traversed by several branches of the pulmonary artery, one of which opened into the cavity, which had a distinct lining membrane, and contained a large coagulum. The lung was enlarged and completely occupied by tubercular deposition; the right lung was also studded with miliary tubercles. The stomach was distended with very dark blood and clots; tubercular deposits in the liver; mesenteric glands enlarged.

*Case* 3.—Fanny B——, æt. 9 months. Was deserted at the gate of the institution eight months ago, had aphonia at that time, and never could cry, although it had no dyspnœa or croupy cough; the aphonia continued up to its death, *which was caused by profuse hæmoptysis.*

The rima glottidis was nearly closed by a fibrinous deposition which occupied the chordæ vocales, and extended into the ventricle of the larynx. In the inferior lobe of the left lung was found a large irregular cavity, into which a branch of the pulmonary artery opened. The whole lung was hypertrophied and occupied by tubercular deposition.

*Remarks.*—The above cases have been considered worthy of being recorded, not only from the great interest which must always be attached to sudden and profuse bleeding from the lungs, as well as from the rare occurrence of hæmoptysis at so early an age, but also because every case of sudden death (particularly in children) which can be explained satisfactorily by the state of the organ after death, is of importance; and lastly, as there are few, if any, accurate accounts of the bleeding vessel having been distinctly traced into the cavity.

The first case is peculiarly interesting, as an instance of what Bayle calls the phthisie ulcereuse. It appears that gangrene had attacked the tubercular cavity, and a portion of the lung had given way, probably from mechanical causes, as there were no appearances of pleuritic inflammation having been set up in the adjacent parts, and Nature had neglected her usual precautionary measure of obliterating the canal of the artery to provide against hæmorrhage.

The appearance of the blood in the stomach in the second case was very striking. It is doubtful whether its black colour was to be attributed solely to the chemical action of the gastric acid, or partly to its being, as it were, pumped directly from the pulmonary artery into the stomach.

The last case presented some points of peculiar interest.

1st. The extreme youth of the infant.

2nd. The existence of vegetations of a peculiar character nearly closing the glottis, and yet not causing any stricture, dyspnœa, nor appearing to affect in any manner except by producing aphonia.

3rd. The granular appearance of the cavity, not lined by any membrane.

4th. The hypertrophy of the lung in which the cavity was formed. This state is oftener met with in connexion with tubercular development than is generally supposed, and if, on investigation, found to be the case in many instances, must make us qualify the proposition, that atrophy of the lung always attends the earlier stages of tubercle, and that a contraction of the chest must necessarily follow.

